# Patient‐Reported Outcomes for Patients With Metastatic NSCLC Treated at an Academic Medical Center, 2017–2021

**DOI:** 10.1002/cam4.71111

**Published:** 2025-08-01

**Authors:** Loretta A. Williams, Cai Xu, Darcy A. Ponce, Qiuling Shi, Melissa L. Santorelli, Thomas Burke, Mehmet Altan

**Affiliations:** ^1^ Department of Symptom Research The University of Texas MD Anderson Cancer Center Houston Texas USA; ^2^ Value and Implementation Outcomes Research, Oncology Merck & Co., Inc. Rahway New Jersey USA; ^3^ Outcomes Research MRL, Merck & Co., Inc. Rahway New Jersey USA; ^4^ Department of Thoracic/Head and Neck Medical Oncology The University of Texas MD Anderson Cancer Center Houston Texas USA

**Keywords:** advanced lung cancer, first‐line treatment, patient‐reported outcomes, symptom burden, symptom interference, symptoms

## Abstract

**Background:**

Understanding the symptom burden experienced by patients with cancer can enable appropriate supportive care. Our aim was to describe patient‐reported outcomes (PROs) for patients with metastatic NSCLC initiating first‐line (1L) systemic therapy under usual care at a large academic center.

**Methods:**

Patients eligible for this prospective observational study were ≥ 18 years old when initiating 1L systemic therapy for stage IV NSCLC from January 1, 2017 to December 31, 2020. Patients completed two PRO questionnaires before 1L therapy initiation (baseline) and every ~6 weeks at imaging visits thereafter: the MD Anderson Symptom Inventory for lung cancer (MDASI‐LC) and EuroQol EQ‐5D‐5L. Study follow‐up ended on June 30, 2021.

**Results:**

The 609 eligible patients (median age, 63 years; range, 24–87; 51% men) received 1L chemotherapy plus immunotherapy (38%), targeted therapy (29%), chemotherapy (19%), or immunotherapy (13%). The five most severe MDASI‐LC symptoms among all assessments were fatigue, pain, shortness of breath, disturbed sleep, and dry mouth; 17%–32% of assessments were rated as moderate or severe. Of all assessments, 39% recorded moderate to severe symptom interference with physical aspects of daily life, and 22% reported moderate to severe interference with affective aspects. Symptom and interference subscale scores generally declined from baseline to 102 weeks overall and for the four 1L regimens.

**Conclusion:**

Our findings confirm previous findings that patients with a new diagnosis of metastatic NSCLC experience a moderate symptom burden. More research is needed to identify predictors and causes of this symptom burden so that it can be effectively addressed.

## Graphical abstract

This prospective observational study at an academic center enabled a detailed description of the symptom burden before and during first‐line systemic therapy for 609 patients with metastatic NSCLC. Study findings align with prior research indicating that patients with a new diagnosis of metastatic NSCLC experience a moderate symptom burden.

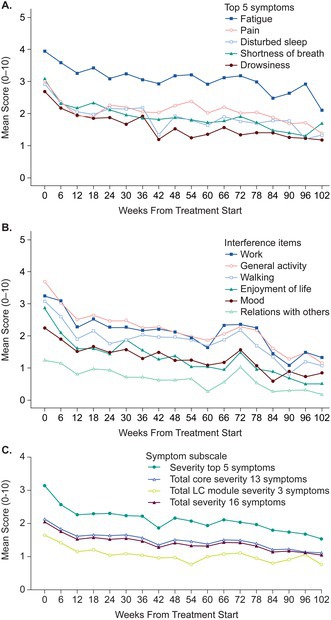

## 1 Introduction

The importance of assessing the patient perspective using patient‐reported outcomes (PROs), including symptom burden and health‐related quality of life (HRQoL), is increasingly recognized for conditions requiring long‐term therapy, such as cancer [1, 2, 3]. An understanding of the symptom burden that patients are experiencing from their cancer, as well as from cancer therapy, can enable health care providers to provide appropriate supportive care [4]. Moreover, recent clinical trial evidence suggests that the use of PROs for symptom monitoring may have prognostic value and provide clinical benefits, although further study is needed [5, 6, 7, 8].

The treatment of advanced and metastatic non‐small cell lung cancer (NSCLC) is rapidly evolving with the availability of immunotherapies and targeted therapies. Assessments of PROs, including symptom burden and HRQoL [9, 10, 11], are usually secondary or exploratory endpoints in cancer clinical trials and often reported much later than the efficacy results [12, 13, 14, 15]. The US Food and Drug Administration (FDA) published guidance in 2009 regarding use of PROs to support labeling claims [16]; however, a subsequent review concluded that the lack of clear PRO research objectives and analytic standardization were key issues limiting the interpretation of PROs collected in pivotal lung cancer trials submitted to the FDA as part of the drug approval process from 2008 to 2017 [14]. Information from real‐world settings can supplement and complement clinical trial results and potentially improve our understanding of the disease burden of NSCLC and of therapy‐related symptoms.

The aim of this observational single‐center study was to describe PROs for patients with metastatic NSCLC initiating first‐line (1L) systemic therapy in the context of usual care at a large academic center. The PRO data, including symptom scores and self‐rated overall health status, were collected in a prospective fashion, alongside data from patient charts, as part of a noninterventional study conducted at MD Anderson Cancer Center (MDACC). The testing, treatment, and clinical outcome measures have been reported separately [17].

## 2 Methods

### 2.1 Setting and Patients

Approval of the protocol for the prospective phase of this observational study was provided by The University of Texas MDACC Institutional Review Board (IRB# BS99‐094; Measuring the Symptom Distress of Cancer Patients), and patients provided written consent, in addition to written consent for the GEMINI‐Moonshot Project (A prospective database for patients with lung cancer incorporating collection of tissue and clinical information; MDACC IRB# PA13‐0589).

Patients 18 years and older initiating 1L systemic therapy for stage IV NSCLC from January 1, 2017 to December 31, 2020, and who consented to PRO collection were eligible for study inclusion. Their clinical data had to be available in the GEMINI database, and patients had to complete at least one PRO assessment to remain eligible for the PRO analyses. Those who came to MDACC solely one time for a second opinion were excluded, as were those enrolled in a clinical trial of 1L immunotherapy. However, patients already in the study who enrolled in a subsequent clinical trial (second‐line or greater) continued on study. Patient follow‐up was conducted through June 30, 2021, and patients were followed regardless of disease status or treatment change until they asked to be removed from the study, died, or were lost to follow up for > 6 months. We note that COVID restrictions were put in place at MDACC on March 12, 2020 (discussed further below).

### 2.2 Patient‐Reported Outcome Measures

Eligible patients completed two PRO questionnaires before 1L therapy initiation (baseline) and at MDACC imaging visits for treatment response assessment thereafter: the MD Anderson Symptom Inventory (MDASI) for lung cancer (MDASI‐LC) [1, 18, 19] and the EuroQol Group's EQ‐5D‐5L [20]. The MDASI‐LC, which takes less than 5 min to complete, assesses 3 symptoms specific to lung cancer (coughing, constipation, and sore throat), in addition to the 13 MDASI core symptoms and 6 MDASI symptom interference items. The 13 MDASI core symptoms are pain, fatigue, nausea, disturbed sleep, distress, shortness of breath, difficulty remembering, lack of appetite, drowsiness, dry mouth, sadness, vomiting, and numbness/tingling. The 6 MDASI interference items assess symptom interference with physical aspects of daily life (work, general activity, and walking [WAW] subscale) and with affective aspects of daily life (relations with others, enjoyment of life, and mood [REM] subscale) [19].

The severity of symptoms at their worst in the prior 24 h is assessed by the MDASI on a scale of 0 (not present) to 10 (as bad as you can imagine). The severity of symptom interference with normal daily activities in the prior 24 h is rated on a scale of 0 (did not interfere) to 10 (interfered completely) [10, 18, 19]. In line with a prior study at our institution, we categorized symptoms as mild when rated > 0–4, moderate when rated > 4–< 7, and severe when rated ≥ 7 on the MDASI scale of 0–10 [21]. We categorized symptom interference as mild when rated > 0–< 2, moderate when rated ≥ 2–5, and severe when rated > 5 [22].

The EQ‐5D‐5L is a PRO instrument scored by patients on a 5‐point scale from “no problem” to “extreme problem” (see Appendix S1). We used the US‐based scoring algorithm to generate US population preference‐weighted health index scores [23]. The second page of the EQ‐5D‐5L comprises the EQ visual analogue scale (EQ VAS) on which patients self‐rate their health from 0 (worst) to 100 (best) [20].

Real‐world compliance rates at baseline and subsequent timepoints were calculated for each PRO instrument as the percentage of patients who completed an assessment (defined as completing at least 50% of the questionnaire items) among those who were expected to complete at each timepoint.

### 2.3 Analyses

We used summary statistics to describe patient characteristics overall and by 1L treatment regimen categorized as chemotherapy, chemotherapy plus immunotherapy, immunotherapy, or targeted therapy. Study follow‐up of individual patients ended if/when lost to follow up for more than 6 months, on the date of death, or on June 30, 2021, whichever occurred first.

The first PRO analytic timepoint was at “baseline” defined as ±3 weeks from 1L therapy initiation, with “true baseline” limited to the 3 weeks preceding 1L therapy initiation. After 1L therapy initiation, PRO analytic timepoints occurred every 6 weeks based on an average 6‐week interval for treatment response assessment. Timepoints included an analytic window of ±3 weeks, for a total window of 6 weeks around each expected imaging visit (Table S1).

To explore longitudinal trends, mean scores for individual symptom severity and symptom interference, and for several subscales, were summarized in tables and graphs. Only non‐missing values were retained, with no imputation methods applied for missing PRO data. We summarized the means at each assessment timepoint up to week 102, after which the number of patients still active on the study became too small for statistical relevance. The primary estimand in this study was descriptive, aiming to characterize the average symptom burden and HRQoL over time from the initiation of 1L therapy. We adopted an estimand framework to address intercurrent events: for non‐fatal events such as treatment change or disease progression, we included all available PRO assessments regardless of these changes; for death, we applied a while‐alive strategy by including only PRO assessments collected before the date of death, with no imputation or extrapolation beyond that point.

The MDASI‐LC symptom severity and interference scores, EQ‐5D‐5L weighted scores, and EQ VAS scores were retrospectively examined longitudinally using mixed‐effect modeling by 1L treatment response as determined by the clinician (complete/partial response, stable disease, or progressive disease) and by 1L treatment type. Parameter estimates were estimated from the final “best” restricted maximum likelihood model, as maximum likelihood may underestimate the variance of the random effects. *p*‐values were calculated using Kenward‐Roger standard errors and degrees of freedom, and *p*‐values < 0.05 were considered significant.

All eligible patients were included in the analyses, and no formal sample size calculation was conducted for this descriptive study. Statistical analyses were conducted using SAS version 9.4 (SAS Institute Inc., Cary, NC, US) and R package (v4.2.1, R Core Team, 2022).

## 3 Results

### 3.1 Patients

**FIGURE 1:**
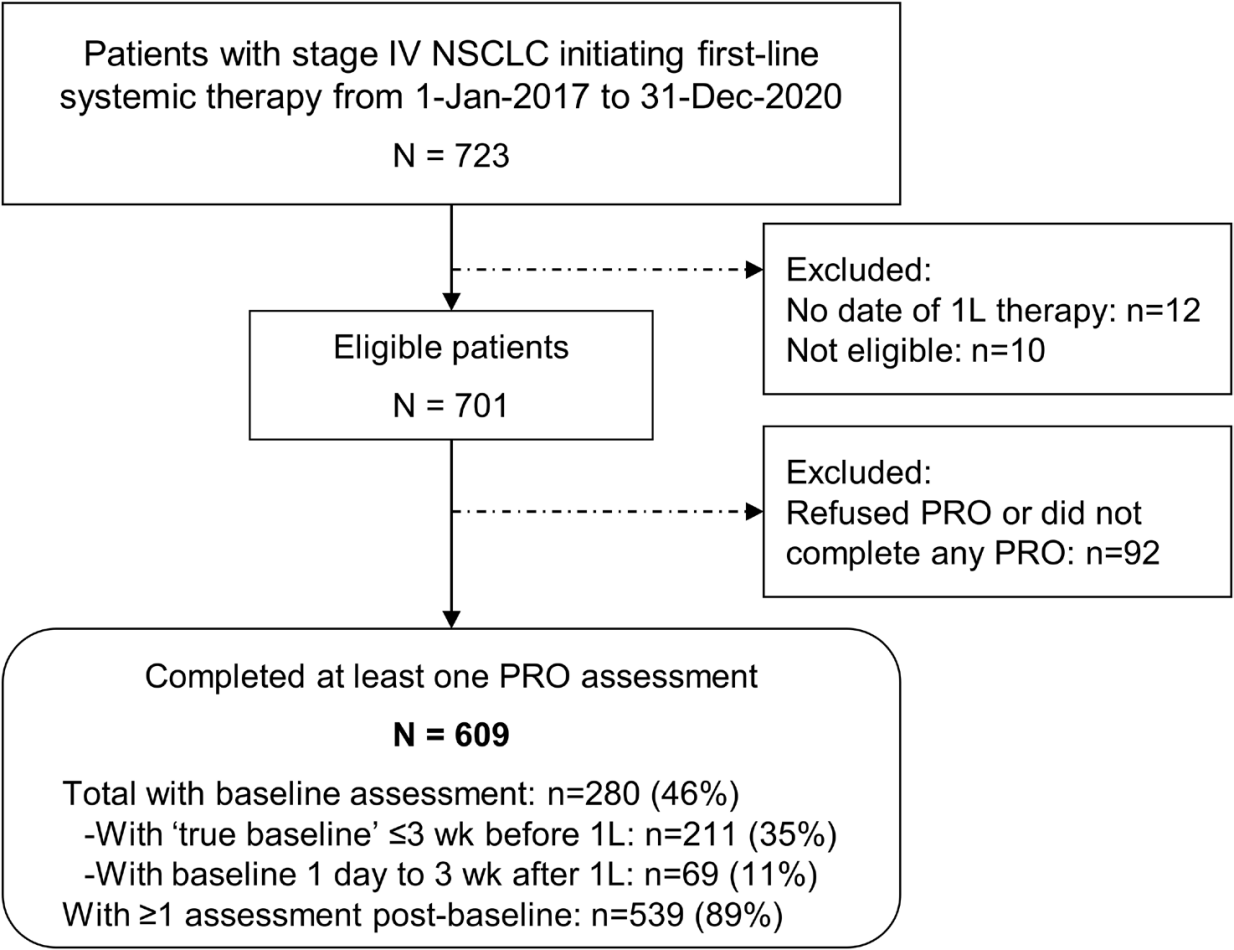
Identification of patients included in the analyses of patient‐reported outcomes. 1L, first‐line therapy; PRO, patient‐reported outcome; wk, weeks.

A total of 609 patients with stage IV NSCLC consented to PRO data collection and completed at least one PRO assessment. The 280 patients (46%) with baseline PRO data included 211 patients who completed a baseline assessment before starting 1L therapy (“true baseline”) and 69 patients who completed an assessment within 3 weeks after starting 1L therapy (Figure 1), while 329 patients were already receiving 1L therapy (i.e., initiated 1L > 3 weeks before study entry) and thus were not eligible for the baseline PRO assessment.

All patients answered both the MDASI‐LC and the EQ‐5D‐5L when they answered either PRO instrument at any assessment timepoint. Thus, the compliance rates for the MDASI‐LC and EQ‐5D‐5L were identical. At 6 weeks after 1L therapy initiation, PRO instrument compliance rates were 61% and then gradually fell during the 6 months to 48%, subsequently increasing to 59% at 54 weeks (Table S2). Compliance rates were based on patients active in the study at each timepoint and were not restricted to patients available for PRO assessments at that timepoint; therefore, patients who did not have a scheduled MDACC imaging visit at a given timepoint, and thus were not given an opportunity to complete PRO assessments, were included in the denominator for the compliance calculations.

The median age of 609 eligible patients was 63 years (range, 24–87 years); 313 patients (51%) were men; 482 (79%) were White; and 359 (59%) were current or former smokers (Table 1). Most patients with recorded performance status had an ECOG PS of 0 or 1 (387; 87%), and, for most, their first NSCLC diagnosis was at stage IV (529; 87%). Table S3 depicts patient characteristics by baseline PRO assessment status.

**TABLE 1:** Patient characteristics overall and by first‐line treatment regimen.

Characteristic	All patients (*N* = 609)	Treatment regimen (*N* = 607)^a^			
		Chemo + IO (*n* = 232)	Targeted therapy (*n* = 176)	Chemo (*n* = 117)	IO (*n* = 82)
Sex, No. (%)					
Male	313 (51.4)	121 (52.2)	67 (38.1)	77 (65.8)	47 (57.3)
Female	296 (48.6)	111 (47.8)	109 (61.9)	40 (34.2)	35 (42.7)
Age, median (range)	63 (24–87)	65 (26–87)	61 (24–83)	64 (37–82)	67 (41–87)
Race, No. (%)					
White	482 (79.1)	183 (78.9)	132 (75.0)	96 (82.1)	69 (84.2)
Black	64 (10.5)	32 (13.8)	10 (5.7)	14 (12.0)	8 (9.8)
Asian	40 (6.6)	9 (3.9)	26 (14.8)	2 (1.7)	3 (3.7)
Other	23 (3.8)	8 (3.4)	8 (4.5)	5 (4.3)	2 (2.4)
Ethnicity, No. (%)					
Hispanic	40 (6.6)	13 (5.6)	14 (8.0)	7 (6.0)	6 (7.3)
Non‐Hispanic	569 (93.4)	219 (94.4)	162 (92.0)	110 (94.0)	76 (92.7)
Smoking history, No. (%)					
Current smoker	63 (11.5)	34 (17.7)	3 (1.8)	18 (16.5)	8 (10.5)
Former smoker	296 (54.2)	114 (59.4)	51 (30.5)	76 (69.7)	53 (69.7)
Nonsmoker	187 (34.2)	44 (22.9)	113 (67.7)	15 (13.8)	15 (19.7)
Missing	63	40	9	8	6
BMI, mean (SD), kg/m^2^	27.3 (14.0)	27.1 (6.3)	25.7 (5.2)	26.2 (5.6)	26.8 (5.7)
ECOG PS, No. (%)					
0–1	387 (87.4)	147 (87.0)	118 (91.5)	75 (89.3)	47 (77.0)
2	45 (10.2)	16 (9.5)	9 (7.0)	7 (8.3)	13 (21.3)
3	11 (2.5)	6 (3.6)	2 (1.6)	2 (2.4)	1 (1.6)
Missing	166	63	47	33	21
Stage IV at initial diagnosis, No. (%)	529 (86.9)	206 (88.8)	155 (88.1)	97 (82.9)	70 (85.4)

*Note:* Percentages may not add up to 100 because of rounding.

Abbreviations: BMI, body mass index; Chemo, chemotherapy; ECOG PS, Eastern Cooperative Oncology Group performance status; IO, immunotherapy.

^a^ Patients who received anti‐vascular endothelial growth factor (anti‐VEGF) therapy along with chemotherapy plus immunotherapy (*n* = 1), targeted therapy (*n* = 3), or chemotherapy (*n* = 5) were combined with those respective groups; 2 patients who received anti‐VEGF monotherapy were not included in the treatment regimen groups.

Chemotherapy plus immunotherapy was the largest 1L treatment group, including 232 patients (38%) (Table 1). The targeted therapy group included 176 patients (29%), while 117 patients (19%) received chemotherapy, and 82 patients (13%) received therapy with a PD‐(L)1 inhibitor (immunotherapy) as monotherapy or in combination with a second immune checkpoint inhibitor (ICI). Patients who received targeted therapy were more often female, younger (median age, 61), and Asian, as well as more often a nonsmoker than those who received the other three regimen types. The 82 patients who received immunotherapy (without chemotherapy) were more often older (median age, 67) and more often had an ECOG PS of ≥ 2 than those who received the other regimens (Table 1).

### 3.2 Patient‐Reported Outcomes on the MDASI‐LC

**FIGURE 2:**
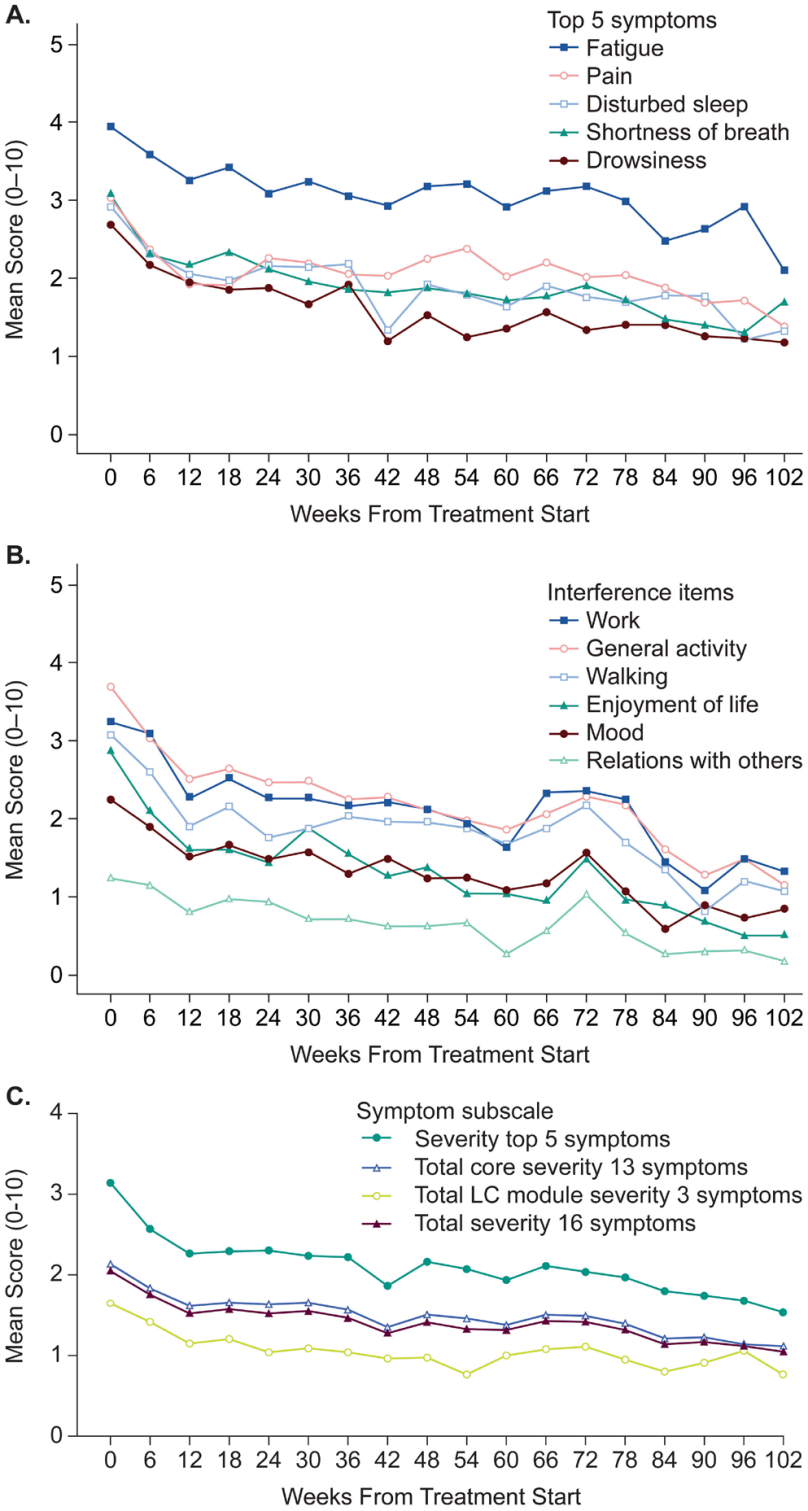
Longitudinal plots of the mean MD Anderson Symptom Inventory for lung cancer (MDASI‐LC) scores from week 0 (baseline) to week 102 after first‐line therapy initiation of (A) the five most severe symptoms at baseline (fatigue, pain, disturbed sleep, shortness of breath, drowsiness), (B) the 6 individual interference items, and (C) the symptom subscales of the 5 most severe symptoms overall (fatigue, pain, shortness of breath, disturbed sleep, dry mouth), all 16 MDASI‐LC symptoms, the 13 core MDASI symptoms, and the 3 lung cancer (LC) module symptoms. On the MDASI, patients rate the severity of symptoms at their worst in the prior 24 h from 0 (not present) to 10 (as bad as you can imagine) and the severity of symptom interference with normal daily activities in the prior 24 h from 0 (did not interfere) to 10 (interfered completely).

The five most severe symptoms reported at baseline (i.e., with the highest mean scores) by the 280 patients with baseline assessments were fatigue, shortness of breath, pain, disturbed sleep, and drowsiness, with 16%–27% of patients rating these symptoms as severe (Table 2, Figure 2). The 3 interference items related to physical aspects of daily life (work, general activities, walking) were rated as severe by 35%–41% of patients, and the 3 items related to affective aspects (relations with others, enjoyment of life, mood) were rated as severe by 12%–31% of patients; the WAW subscale thus had greater means at baseline than the REM subscale (Table 2). These findings were similar among the 211 patients with “true baseline” assessments (*t*‐test *p* ≤ 0.05 for all comparisons of item and subscale means between all baseline and true baseline groups; data not shown).

**TABLE 2:** MDASI‐LC scores at baseline for 280 patients with PRO assessments within 3 weeks of initiating first‐line therapy.

Symptom severity items (rank order)	*N*	Mean	SD	LCL	UCL	Ratings in each score category, %^a^			
						0	> 0–4	> 4–< 7	≥ 7
Fatigue	280	3.95	3.14	3.58	4.32	22.5	32.5	18.21	26.79
Shortness of breath	280	3.09	3.11	2.72	3.45	32.86	36.07	11.43	19.64
Pain	280	3.03	3.17	2.66	3.40	37.86	30.00	11.78	20.36
Disturbed sleep	279^b^	2.92	3.30	2.54	3.31	42.50	25.71	12.86	18.57
Drowsiness	280	2.70	2.93	2.35	3.04	36.07	37.14	10.36	16.43
Coughing	280	2.52	2.92	2.17	2.86	38.21	39.64	8.21	13.93
Dry mouth	279^b^	2.39	3.08	2.03	2.75	45.71	32.14	7.50	14.29
Lack of appetite	280	2.23	2.93	1.89	2.58	50.00	26.07	10.72	13.21
Distress	280	2.17	2.74	1.85	2.49	45.71	34.64	9.64	10.00
Constipation	280	1.72	2.67	1.40	2.03	58.57	24.29	8.21	8.93
Difficulty remembering	279^b^	1.51	2.37	1.23	1.78	54.64	32.50	8.93	3.57
Sadness	280	1.44	2.15	1.18	1.69	53.57	36.43	5.71	4.29
Numbness/tingling	280	1.06	2.23	0.79	1.32	73.57	17.14	3.58	5.71
Nausea	280	1.00	2.22	0.74	1.26	74.64	14.29	5.71	5.36
Sore throat	280	0.73	1.85	0.51	0.95	78.93	14.64	2.86	3.57
Vomiting	280	0.33	1.22	0.18	0.47	89.64	7.50	1.43	1.43

Symptom interference	*N*	Mean	SD	LCL	UCL	0	> 0–< 2	≥ 2–5	> 5
General activity	280	3.69	3.51	3.27	4.10	34.29	4.64	19.64	41.43
Work	280	3.25	3.65	2.82	3.68	42.86	6.43	16.07	34.64
Walking	280	3.08	3.36	2.68	3.47	40.71	7.86	16.43	35.00
Enjoyment of life	280	2.86	3.31	2.47	3.25	42.50	9.29	16.78	31.43
Mood	280	2.25	2.74	1.93	2.58	45.00	8.57	22.86	23.57
Relations with others	280	1.24	2.21	0.98	1.50	65.36	8.93	13.57	12.14

Symptom subscales at baseline^c^	*N*	Mean	SD	LCL	UCL	0	> 0–4	> 4–< 7	≥ 7
Severity top 5 items	280	3.14	2.31	2.87	3.41	8.21	56.79	29.64	5.36
Total severity	280	2.05	1.53	1.87	2.23	3.21	86.07	10.36	0.36
Total core severity	280	2.14	1.67	1.94	2.34	3.90	82.10	13.90	0.80
Total LC module severity	280	1.65	1.59	1.47	1.84	22.5	70.00	7.14	0.36

Interference subscales	*N*	Mean	SD	LCL	UCL	0	> 0– < 2	≥ 2–5	> 5
Total interference	280	2.73	2.55	2.43	3.03	18.21	29.65	30.71	21.43
WAW	280	3.34	3.07	2.98	3.70	23.21	18.58	25.71	32.5
REM	280	2.12	2.37	1.84	2.39	31.07	27.14	25.72	16.07

Abbreviations: LC, lung cancer; LCL, lower 95% confidence interval limit; REM, relations with others, enjoyment of life, mood; UCL, upper 95% confidence interval limit; WAW, work, general activities, walking.

^a^ Symptom severity for a score of 1–4 was considered mild; > 4, moderate to severe; and ≥ 7, severe. Interference severity for a score of > 0 was considered mild to severe; ≥ 2, moderate to severe; and > 5, severe.

^b^ Data missing for one patient (0.36%).

^c^ For the symptom subscales at baseline, the “severity top 5 items” at baseline were fatigue, pain, shortness of breath, disturbed sleep, and drowsiness. “Total severity” included the 13 MDASI items plus the 3 lung cancer‐specific items; “total core severity” included the 13 MDASI items, and “total LC module severity” included the 3 lung cancer‐specific items.

During the course of the study, mean scores for each symptom were ≤ 3.1 (Table 3). The most severe symptoms reported overall were fatigue, pain, shortness of breath, disturbed sleep, and dry mouth. Fatigue was rated as moderate or severe in 32% of assessments, pain in 21%, shortness of breath in 18%, disturbed sleep in 18%, and dry mouth in 17% (Table 3; Tables S4–S6). Symptom interference with general activity was rated as moderate or severe in 42% of assessments, with work in 40% of assessments, and with walking in 35%.

**TABLE 3:** MDASI‐LC mean scores during the study: All assessments combined.

Symptom	Obs, No.	Mean	SD	% Moderate (> 4– < 7)^a^	% Severe (≥ 7)^a^
Fatigue	3599	3.10	2.90	16.0	16.1
Pain	3602	2.08	2.78	10.2	10.7
Shortness of breath	3598	1.95	2.61	9.3	8.6
Disturbed sleep	3598	1.88	2.70	8.2	9.6
Dry mouth	3595	1.78	2.65	7.8	8.8
Drowsiness	3598	1.61	2.36	7.1	6.5
Coughing	3599	1.47	2.30	5.8	5.9
Lack of appetite	3597	1.45	2.47	7.1	7.0
Difficulty remembering	3600	1.42	2.21	6.1	5.0
Constipation	3599	1.22	2.31	4.9	5.9
Distress	3601	1.19	2.17	4.8	4.9
Numbness/tingling	3601	1.18	2.19	5.3	4.8
Sadness	3598	0.83	1.83	3.4	3.2
Nausea	3596	0.77	1.85	3.1	3.2
Sore throat	3597	0.40	1.30	1.4	1.5
Vomiting	3599	0.29	1.26	0.7	1.6

Symptom interference	Obs, No.	Mean	SD	% Moderate (≥ 2–5)	% Severe (> 5)
General activity	3599	2.20	3.01	26.1	15.6
Work	3600	2.11	2.99	24.4	15.2
Walking	3599	1.86	2.82	22.1	13.0
Enjoyment of life	3596	1.34	2.43	17.8	8.7
Mood	3596	1.30	2.25	21.1	6.9
Relations with others	3599	0.67	1.68	10.8	3.5

Symptom subscales^b^	Obs, No.	Mean	SD	% Moderate (> 4–< 7)	% Severe (≥ 7)
Severity top 5 items	3584	2.15	1.92	14.2	2.4
Total severity	3555	1.41	1.31	4.8	0.1
Total core severity	3565	1.50	1.41	6.4	0.3
Total LC module severity	3591	1.03	1.38	3.9	0.4

Interference subscales	Obs, No.	Mean	SD	% Moderate (≥ 2–5)	% Severe (> 5)
Total interference	3586	1.58	2.05	22.3	8.4
WAW	3596	2.06	2.64	23.9	14.8
REM	3590	1.10	1.84	16.7	5.3

Abbreviations: LC, lung cancer; No., number; Obs, observations; REM, relations with others, enjoyment of life, mood; WAW, work, general activities, walking.

^a^ Percentages calculated after removing the missing values. Symptom severity for a score of > 4 to < 7 was considered moderate, and ≥ 7, severe. Interference severity for a score of ≥2 to 5 was considered moderate, and > 5, severe.

^b^ For the symptom subscales, the severity top 5 items were fatigue, pain, shortness of breath, disturbed sleep, and dry mouth. “Total severity” included the 13 MDASI items plus the 3 lung cancer‐specific items; “total core severity” included the 13 MDASI items, and “total LC module severity” included the 3 lung cancer‐specific items.

The mean scores for the five most severe symptoms identified at baseline (fatigue, shortness of breath, pain, disturbed sleep, and drowsiness) showed a noticeable decrease in severity during the first 3 months after 1L therapy initiation (Figure 2A), with a similar pattern noted for the mean scores of the 6 symptom interference items (Figure 2B) and the mean symptom subscale scores (Figure 2C).

**FIGURE 3:**
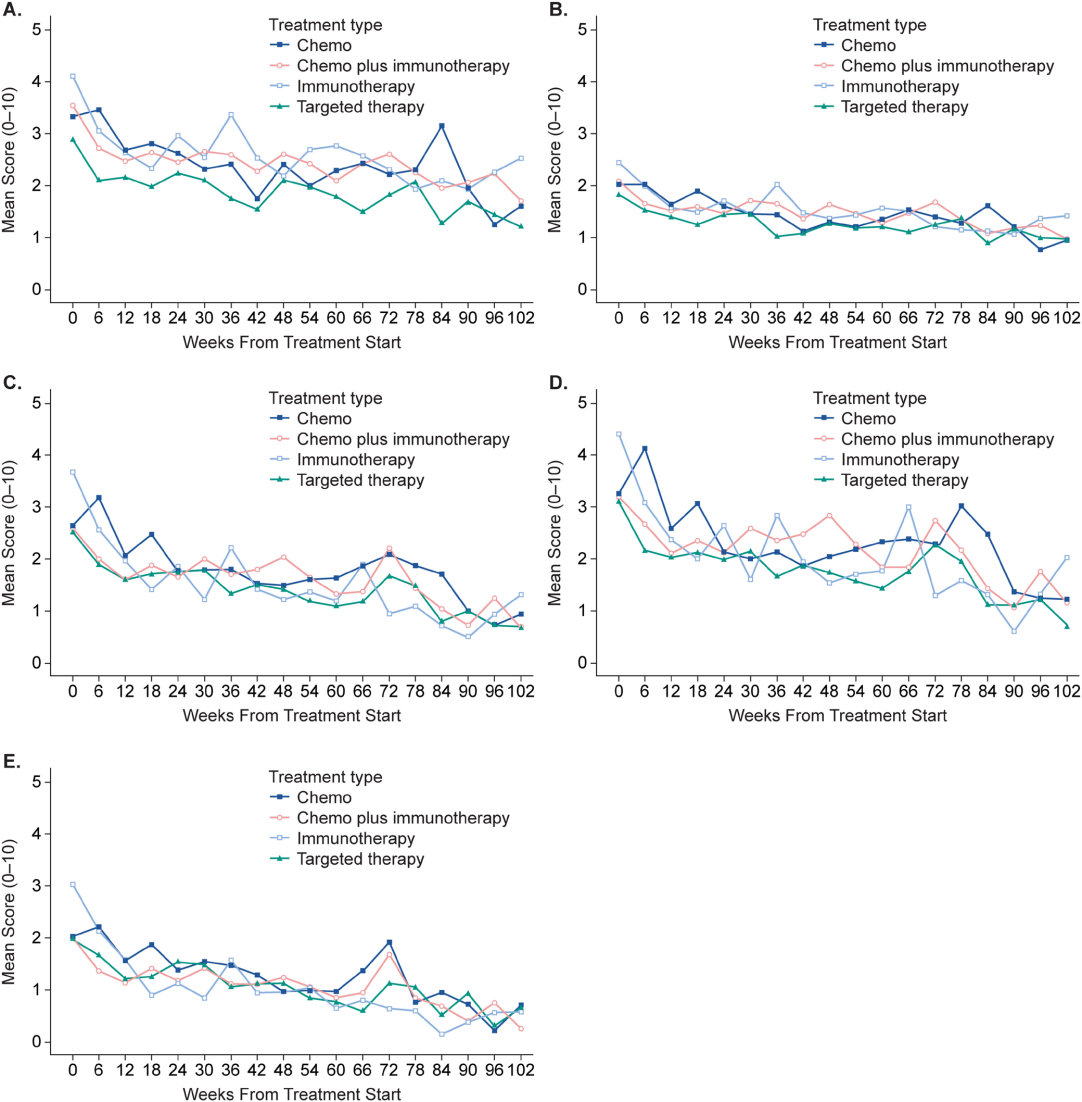
Mean MD Anderson Symptom Inventory for lung cancer (MDASI‐LC) scores from the baseline assessment to week 102, depicted by first‐line treatment regimen: (A) the five most severe symptoms overall (fatigue, pain, shortness of breath, disturbed sleep, drowsiness), (B) subscale scores of all 16 MDASI‐LC symptom items, (C) subscale score of all 6 MDASI‐LC interference items, (D) subscale score of all 3 MDASI‐LC physical interference items (WAW – work, general activities, walking), and (E) subscale score of all 3 MDASI‐LC affective interference items (REM – relations with others, enjoyment of life, mood). Patient numbers vary for each timepoint; all available scores were included. Patient ratings on the MDASI scale are from 0 (best) to 10 (worst).

The symptom and interference subscale scores also generally declined from baseline to 102 weeks after the start of 1L therapy for the four 1L regimens (Figure 3). At baseline, patients receiving different 1L regimens had observable but nonsignificant (*p* = 0.058) differences in the symptom burden subscale score, measured as the mean of the five most severe symptoms (fatigue, pain, shortness of breath, disturbed sleep, and drowsiness; Figure 3A). Patients receiving immunotherapy had the greatest symptom burden at baseline (mean score, 3.82; SD 2.25), followed by chemotherapy plus immunotherapy (mean, 3.28; SD 2.31) and chemotherapy (mean, 3.09; SD 2.24), while those receiving targeted therapy had the lowest burden at baseline (mean, 2.64; SD 2.31). Over the course of 102 weeks, increases from baseline in the means for this subscale were evident among some 1L regimens that differed from those of other regimens, such as a significant increase (*p* = 0.003) in this symptom burden with immunotherapy at about 36 weeks, while the symptom burden increase with chemotherapy at about 84 weeks was observable but nonsignificant (*p* = 0.094) in the five most severe symptoms (Figure 3A). A modest increase in symptoms was evident with chemotherapy plus immunotherapy at 72 weeks but was nonsignificant (*p* = 0.332; Figure 3A). None of these increases reached the severity level observed at baseline in the most severe baseline symptoms subscale for the respective treatment regimens.

### 3.3 Patient‐Reported Outcomes on the EQ‐5D‐5L

At baseline, the mean EQ‐5D‐5L preference weighted score was 0.82 (SD, 0.21; with maximum [best] score of 1). Over the 2 years of follow‐up, the EQ‐5D‐5L weighted scores fluctuated but ended close to the baseline value (mean, 0.84; SD, 0.19), with a mean score for all observations of 0.82 (SD, 0.22). The lowest mean score of 0.75 (SD, 0.30) was recorded at 78 weeks, within 4 weeks of an increase occurring in the symptom interference scores.

The EQ VAS score increased over the 2 years of follow‐up from a baseline mean of 72.49 (SD, 19.94) to a mean of 78.48 (SD, 17.54) at 102 weeks. The mean VAS score for all assessments was 75.11 (SD, 19.43).

**FIGURE 4:**
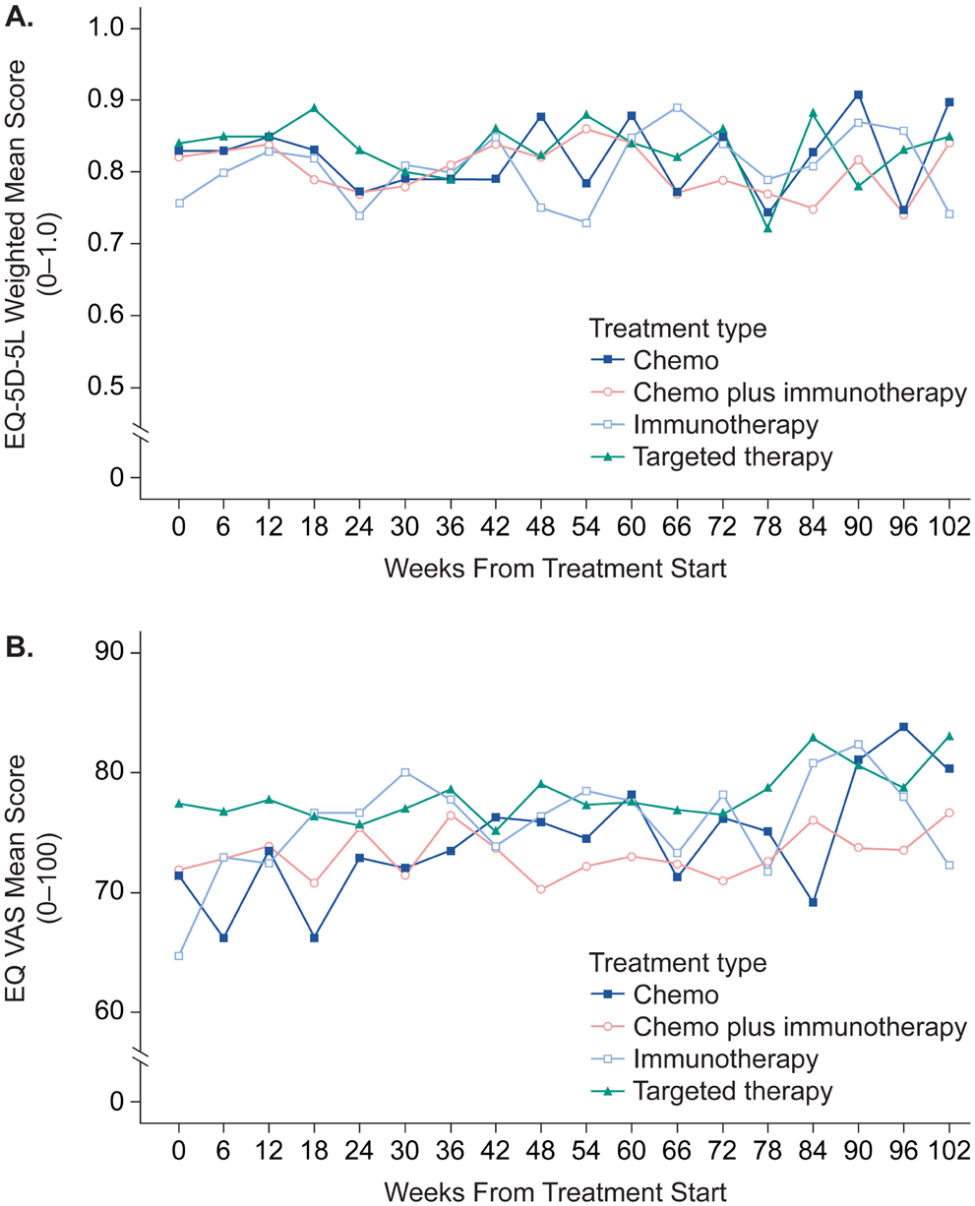
Means from the baseline assessment to week 102, depicted by 1L treatment regimen: (A) EQ‐5D‐5L weighted scores (mobility, self‐care, usual activities, pain/discomfort, and anxiety/depression scored by patients from “no problems” to “extreme problems,” with 1 being best weighted score), and (B) EQ visual analogue scale (EQ VAS) scores, scored from 0 (the worst health you can imagine) to 100 (the best health you can imagine). Chemo, chemotherapy; VAS, visual analogue scale.

The EQ‐5D‐5L preference weighted scores and EQ VAS scores from baseline assessment to week 102 by 1L treatment regimen are depicted in Figure 4.

### 3.4 Symptom Severity Assessed Longitudinally by Mixed‐Effects Modeling

The numbers of patients in each category of the two parameters investigated for effects on PROs (best 1L treatment response as determined by the clinician and treatment type) are reported in Table S7. In brief, patients who experienced disease progression on 1L therapy reported significantly more severe symptoms over time (except for numbness/tingling), as well as more symptom interference with all 6 items than patients with response to 1L therapy, while individual MDASI‐LC scores were not significantly different between patients with stable disease versus response on 1L therapy.

There were no significant differences over time in the EQ‐5D‐5L weighted scores. The EQ VAS scores were significantly better over time for patients with response to 1L therapy than for patients with disease progression and for patients receiving targeted therapy than for those receiving chemotherapy (complete results reported in Table S8).

## 4 Discussion

This prospective observational study enabled a comprehensive, detailed description of the symptom burden before and during 1L systemic therapy for 609 patients with metastatic NSCLC. On the MDASI‐LC questionnaire, four of the five most severe symptoms (greatest mean scores) at baseline remained over the course of the study among the five most severe symptoms overall (fatigue, pain, shortness of breath, and disturbed sleep), while dry mouth replaced baseline drowsiness as the fifth most severe overall, with the percentages of moderate to severe symptom reports ranging from 17% (dry mouth) to 32% (fatigue). On the WAW and REM subscales, 39% of all assessments recorded moderate to severe symptom interference with physical aspects of daily life (work, general activities, walking), and 22% reported moderate to severe interference with affective aspects (relations with others, enjoyment of life, and mood).

The most severe symptoms recorded at baseline in this study (fatigue, pain, shortness of breath, disturbed sleep, and drowsiness) were mostly aligned with those reported in a prior study at our center of patients with first diagnosis of lung cancer (i.e., fatigue, pain, disturbed sleep, distress, and shortness of breath) [21]. In the latter study, which included 289 patients (63%) with advanced lung cancer, about 20% of all patients reported those five symptoms as being severe on the MDASI around the time of lung cancer diagnosis, while 16%–27% in the present study rated the five symptoms as being severe at baseline. We observed that patients treated with first‐line immunotherapy in our study were more likely to have an ECOG PS of ≥ 2 and had a greater baseline symptom burden, measured as mean MDASI‐LC score, than those who received other regimens, whereas those who received targeted therapy had the lowest symptom burden.

Other recent studies assessing PROs for patients with lung cancer cannot be directly compared with the present study because of varying study designs and different PRO instruments used [24, 25, 26, 27, 28]. Nonetheless, the conclusions of these studies support the integration of PROs in oncology practice as a means to best understand the patient perspective and enable shared decision‐making. Other reported benefits of collecting PROs for patients with cancer (not limited to lung cancer) include the ability to understand the patient experience directly without clinician interpretation and perspectives [29], with the potential to improve physician‐patient communication, symptom awareness, and HRQoL [30, 31]. The use of electronic PRO (ePRO) tools for monitoring symptoms remotely between clinic visits, being implemented at many institutions, will likely further help in accomplishing these goals [24, 32, 33]. Moreover, in randomized trials and community oncology studies, assessing PROs has been associated with a reduced number of ED visits and hospitalizations [7, 34, 35].

In longitudinal findings of our study, the mean scores of individual items and subscales generally decreased in severity, notably with the greatest decrease observed during the first 3 months after initiation of therapy. These improvements in patients who remained on study were observed over time during the first 2 years of 1L therapy initiation. This finding could be clinically relevant as a possible indicator that many patients can expect to feel better after starting treatment, regardless of treatment type, a finding that could be promising or motivating for patients with a new NSCLC diagnosis. It is important to note that these findings could be confounded by patient self‐selection over time. Patients experiencing disease progression may have discontinued from the study either because of death or dropping out because of worsening physical condition.

We found that progression on 1L therapy in this study was significantly associated with the severity of most MDASI‐LC symptoms over time in longitudinal mixed‐effect modeling. Patients with disease progression while on 1L treatment had significant increases in symptom severity and interference over time, except for numbness/tingling, compared with patients with response to 1L treatment. Instead, patients with stable disease on 1L treatment did not differ in symptom severity and interference from those with disease response.

We observed that there was a subset of patients with metastatic NSCLC reporting symptoms at moderate to severe levels. Factors that were associated with the symptom burden that patients experienced over time included the response to 1L treatment and treatment type, with disease progression associated with a higher symptom burden relative to disease responding to 1L therapy, and targeted therapy associated with a lower symptom burden compared with chemotherapy.

This large study provides a comprehensive description of PROs using the MDASI‐LC and EuroQol EQ‐5D‐5L instruments for patients receiving systemic therapies for metastatic NSCLC. The trend in decreasing symptoms identified early in 1L treatment highlights the patient‐reported changes with treatment in a real‐world clinical setting. This study also contributes to the PRO methods literature by illustrating how we conducted PRO analyses in the context of a real‐world setting where the opportunities for assessment are less controlled than in the clinical trial setting. Use of a while‐alive strategy, including only PRO assessments collected before death, with no imputation or extrapolation beyond that point, ensures that our estimates reflect real‐world symptom experiences while patients were alive, an approach aligned with recommendations for descriptive PRO analyses in settings where death is an informative event [36]. To estimate this longitudinal trajectory, we used likelihood‐based mixed‐effects models, which account for repeated measures within individuals and accommodate unbalanced follow‐up due to dropout or administrative censoring. Missing data were handled using listwise deletion (na.action = na.omit), which is appropriate under the missing at random assumption, as mixed‐effects models rely on observed data without requiring imputation. In mixed‐effects models, the treatment response variable was used post hoc to stratify patients and describe longitudinal changes in PROs within response groups.

We also acknowledge several study limitations. Our analyses were descriptive and exploratory, with no predictive or causal interpretations implied. The study was conducted at a single academic center; therefore, results may not be generalizable to other real‐world settings. The MDASI‐LC and EQ‐5D‐5L questionnaires can be completed in a short time, while the compliance rates were relatively low nonetheless, ranging from 48% to 61% during the first year of follow‐up, despite intensive follow‐up by research staff. This was in part due to the method of calculating compliance rates, where the denominator was defined as the number of “possible” assessments (number of patients active on study) at every 6‐week time span. Many “possible” assessments were not done because patients did not return to MD Anderson for response scans (The PRO assessments were done only when patients returned to MD Anderson and had a response scan.).

These compliance rates could also be explained in part by the onset of the COVID‐19 outbreak, declared a pandemic by the World Health Organization on March 11, 2020, approximately 15 months before the end of the study period (June 30, 2021). With patient recruitment for 1L systemic therapy initiation from January 1, 2017 to December 31, 2020, and patient follow‐up through June 30, 2021, approximately 20% of our recruiting time and 30% of our data collection time was potentially impacted by the COVID‐19 pandemic. Patient visits were severely curtailed during the first year of the pandemic, and telehealth visits that did not allow for response scanning at MD Anderson were instituted. The impact of compliance rates on the outcomes reported is unknown. Moreover, the stresses experienced during the pandemic could also have exacerbated patient symptom experience during this time. Our findings regarding compliance indicate that more research is needed to understand facilitators and barriers for completion of PRO instruments in real‐world clinical settings. As remote symptom monitoring using digital communication methods is predicted to become routine in quality cancer care [33], studies such as this one can inform research and the development of ePRO tools.

In conclusion, our overall findings confirm previous findings that patients with a new diagnosis of metastatic NSCLC experience a moderate symptom burden. More research is needed to identify predictors and causes of this symptom burden so that it can be effectively addressed. Improving the understanding of symptoms associated with lung cancer and different treatment regimens and modalities will improve patient care and aid in individualizing patient therapy.

## Author Contributions

**Loretta A. Williams:** conceptualization (equal), investigation (equal), methodology (equal), project administration (equal), resources (equal), supervision (equal), visualization (equal), writing – original draft (equal), writing – review and editing (lead). **Cai Xu:** data curation (lead), formal analysis (equal), software (lead), validation (equal), visualization (equal), writing – review and editing (equal). **Darcy A. Ponce:** investigation (equal), visualization (equal), writing – review and editing (equal). **Qiuling Shi:** formal analysis (equal), validation (equal), visualization (equal), writing – review and editing (equal). **Melissa L. Santorelli:** conceptualization (equal), methodology (equal), project administration (equal), supervision (equal), visualization (equal), writing – original draft (equal), writing – review and editing (equal). **Thomas Burke:** conceptualization (equal), methodology (equal), visualization (equal), writing – review and editing (equal). **Mehmet Altan:** conceptualization (equal), methodology (equal), resources (equal), visualization (equal), writing – review and editing (equal).

## Conflicts of Interest

Loretta A. Williams received research funding from Bayer, Genentech, and Merck. She received consulting fees from AgilePharma Solutions. Melissa L. Santorelli, Thomas Burke are full‐time employees of Merck Sharp & Dohme LLC, a subsidiary of Merck & Co. Inc., Rahway, NJ, USA, and hold stock of Merck & Co. Inc., Rahway, NJ, USA. Mehmet Altan reports receiving research funding (to institution) from Genentech, Nektar Therapeutics, Merck, GlaxoSmithKline, Novartis, Jounce Therapeutics, Bristol Myers Squibb, Eli Lilly, Adaptimmune, Shattuck Lab, Gilead; Advisory Board: GlaxoSmithKline, Shattuck Lab, Bristol Myers Squibb, AstraZeneca, Insightec; Speaker fees from AstraZeneca, Nektar Therapeutics, SITC; and participation in safety review committee for Nanobiotix‐MDA alliance, Henlius. The other authors have no conflicts of interest to report.

## Supporting information


Data S1.

## Data Availability

The health data used to support the findings of this study are restricted by the MD Anderson Cancer Center Institutional Review Board in order to protect patient privacy. For this reason, data used to support the findings of this study have not been made available.
