# Supplementary material for: Patient‐Reported Outcomes for Patients With Metastatic NSCLC Treated at an Academic Medical Center, 2017–2021
Source: Cancer Med. 2025 Aug 1;14(15):e71111. doi: 10.1002/cam4.71111 (PMC12317105; doi:10.1002/cam4.71111)
Supplement: Supplementary file 1 — Data S1. [file CAM4-14-e71111-s001.docx]

***SUPPLEMENTARY FILE***

**Patient-Reported Outcomes for Patients with Metastatic NSCLC Treated at an Academic Medical Center, 2017–2021**

**SUPPLEMENTARY METHODS**

The EQ-5D-5L is a PRO instrument that includes five dimensions of health status (mobility, self-care, usual activities, pain/discomfort, and anxiety/depression) scored by patients on a 5-point scale from ‘no problems’ to ‘extreme problems.’ We used the US-based scoring algorithm, which was based on the time trade-off technique, to generate US population preference-weighted health index scores (score range, -0.57 to 1, with scores <0 defined as worse than dead and 1 being the best score).^1^ The second page of the EQ-5D-5L comprises the EQ visual analogue scale (EQ VAS) on which patients self-rate their health as ‘The worst health you can imagine’ to ‘The best health you can imagine’ from 0 to 100, respectively.^2^

Real-world compliance rates at baseline and subsequent timepoints were calculated for each PRO instrument as the percentage of patients who completed an assessment (defined as completing at least 50% of the questionnaire items) among those who were expected to complete at each timepoint. The numbers of patients expected to complete at each timepoint were the active patients currently on study, excluding patients who had died, had requested to be removed from study, or were lost to follow up for more than 6 months at that timepoint. Patients not undergoing an MDACC imaging scan at that timepoint were not excluded from the calculation, although they were not asked to complete an assessment.

The 1L regimens were categorized as chemotherapy, chemotherapy plus immunotherapy, immunotherapy, or targeted therapy. Patients who received an anti-vascular endothelial growth factor (anti-VEGF) agent in conjunction with chemotherapy, chemotherapy plus immunotherapy, or targeted therapy were grouped with the respective category, while the two patients who received only anti-VEGF therapy were excluded from the analyses by treatment regimen.

The first PRO analytic timepoint was at ‘baseline,’ defined as ±3 weeks from 1L therapy initiation, with ‘true baseline’ limited to the 3 weeks preceding 1L therapy initiation. After 1L therapy initiation, PRO analytic timepoints occurred every 6 weeks based on an average 6-week interval for treatment response assessment. Timepoints included an analytic window of ±3 weeks, for a total window of 6 weeks around each expected imaging visit (Supplemental Table S1). Thus, for example, the analytic window for the first imaging visit ran from 3 weeks +1 day after 1L therapy initiation to 9 weeks after 1L therapy initiation. If two or more PROs were collected during the same analytic window, only the administration closest to the calculated timepoint day was considered, and only PRO assessments within the analytic window were included for a given timepoint.

**Supplemental TABLE S1** | Analysis windows for patient-reported outcomes (PROs) relative to start of first-line therapy (day 0/baseline).

| **Study day** | **Analysis window lower bound** | **Analysis window upper bound** |
| --- | --- | --- |
| True baseline | ≤3 weeks | Day 0/baseline |
| Baseline | ≤3 weeks | ≥3 weeks |
| Imaging visit 1 | ≥3 weeks +1 day after baseline | ≤9 weeks after baseline |
| Imaging visit 2 | ≥9 weeks +1 day after baseline | ≤15 weeks after baseline |
| Imaging visit 3 | ≥15 weeks +1 day after baseline | ≤21 weeks after baseline |

**SUPPLEMENTARY RESULTS**

**Compliance rates for questionnaires**

The baseline compliance rate for the PRO questionnaires among 609 eligible patients was 45.8%, including all 280 patients with baseline assessments within 3 weeks after first-line therapy initiation. One hundred percent of patients who consented within 3 weeks of beginning 1L treatment completed baseline PROs. At 6 weeks, compliance rates were 60.6% and then gradually fell during the first year to 58.6% at 54 weeks (**Supplemental Table S2**). The compliance rates were calculated based on the number of active patients on the study at each time point. Not all active patients were given the opportunity to respond to the PROs at every time point. Patients who did not have a imaging for response evaluation at that time were not given the opportunity to complete a PROs. Therefore, for these 2 reasons, the compliance rates are artificially low.

All patients answered >50% of the questions in the MDASI and EQ-5D-5L whenever they responded to either PRO questionnaire at any assessment time point; therefore, the compliance rates were identical for the two instruments.

**Supplemental TABLE S2** | Numbers of expected and actual PRO assessments at each timepoint and compliance rates for MDASI-LC and EQ-5D-5L by assessment timepoints.

| **Event** | **Expected, N^b^** | **Actual, N** | **Rate (%)** |
| --- | --- | --- | --- |
| 0 week (Baseline)^a^ | 280 | 280 | 100% |
| 6 week | 579 | 351 | 60.6% |
| 12 week | 550 | 287 | 52.2% |
| 18 week | 519 | 297 | 57.2% |
| 24 week | 491 | 236 | 48.1% |
| 30 week | 429 | 224 | 52.2% |
| 36 week | 396 | 222 | 56.1% |
| 42 week | 360 | 190 | 52.8% |
| 48 week | 334 | 186 | 55.7% |
| 54 week | 307 | 180 | 58.6% |
| 60 week | 277 | 164 | 59.2% |
| 66 week | 255 | 156 | 61.2% |
| 72 week | 231 | 138 | 59.7% |
| 78 week | 213 | 124 | 58.2% |
| 84 week | 199 | 119 | 59.8% |
| 90 week | 185 | 104 | 56.2% |
| 96 week | 172 | 100 | 58.1% |
| 102 week | 151 | 96 | 63.6% |
| 108 week | 141 | 74 | 52.5% |
| 114 week | 136 | 81 | 59.6% |
| 120 week^c^ | 122 | 67 | 54.9% |

^a^329 patients started first-line (1L) treatment >3 weeks before study entry and thus were not eligible for the baseline assessment.

^b^The numbers of patients expected to complete at each timepoint were the active patients currently on study, excluding patients who had died, had requested to be removed from study, or were lost to follow up for >6 months at that timepoint. Patients currently on study who did not have a PRO assessment at an expected timepoint, e.g., because they did not have a scheduled imaging visit at MDACC, were still included in the denominator among those expected to have a PRO assessment.

^c^The database allowed calculation of expected and actual completion up to week 120 of the study.

**Patients**

The median age of 609 eligible patients was 63 years (range, 24–87 years); 313 patients (51%) were men; 482 (79%) were White; and 359 (59%) were current or former smokers (Table 1). Most patients with recorded performance status had an ECOG PS of 0 or 1 (387; 87%), and for most their first NSCLC diagnosis was stage IV (529; 87%). Histologic NSCLC types included adenocarcinoma (n = 500; 82%), large cell neuroendocrine carcinoma (16; 3%), squamous cell carcinoma (68; 11%), adenosquamous carcinoma (3; <1%), sarcomatoid carcinoma (2; <1%), and NSCLC not otherwise specified (NOS: 20; 3%). Supplemental Table S3 depicts patient characteristics by baseline PRO assessment status.

**Supplemental TABLE S3 |** Baseline characteristics by baseline assessment status.

| **Characteristic** | **True baseline**  **(N=211)** | | **Baseline not true^a^**  **(N=69)** | | **Total baseline**  **(true + not true: N = 280)** | | **No baseline**  **(N=329)** | |
| --- | --- | --- | --- | --- | --- | --- | --- | --- |
|  | **N** | **%** | **N** | **%** | **N** | **%** | **N** | **%** |
| Sex |  |  |  |  |  |  |  |  |
| Female | 102 | 48.3 | 26 | 37.7 | 128 | 45.7 | 168 | 50.1 |
| Male | 109 | 51.7 | 43 | 62.3 | 152 | 54.3 | 161 | 48.9 |
| Age (years) |  |  |  |  |  |  |  |  |
| Mean ±SD | 63.9 ±11.8 | – | 64.0 ±10.8 | – | 63.9 ±11.6 | – | 61.9 ±11.1 | – |
| Median (range) | 65 (24–87) | – | 65 (33–86) | – | 65 (24–87) | – | 62 (26–87) | – |
| Ethnicity |  |  |  |  |  |  |  |  |
| Hispanic NOS | 12 | 5.7 | 7 | 10.1 | 19 | 6.8 | 21 | 6.4 |
| Non-Hispanic | 199 | 94.3 | 62 | 89.9 | 261 | 93.2 | 308 | 93.6 |
| Race |  |  |  |  |  |  |  |  |
| Asian NOS | 17 | 8.1 | 1 | 1.5 | 18 | 6.4 | 22 | 6.7 |
| Black | 19 | 9.0 | 10 | 14.5 | 29 | 10.4 | 35 | 10.6 |
| Native Hawaiian | 0 | 0 | 0 | 0 | 0 | 0 | 2 | 0.6 |
| Other | 4 | 1.9 | 3 | 4.4 | 7 | 2.5 | 14 | 4.3 |
| White | 171 | 81.0 | 55 | 79.7 | 226 | 80.7 | 256 | 77.8 |
| Tobacco use |  |  |  |  |  |  |  |  |
| Current smoker | 17 | 8.1 | 10 | 14.5 | 27 | 9.6 | 36 | 10.9 |
| Former smoker | 102 | 48.3 | 31 | 44.9 | 133 | 47.5 | 163 | 49.5 |
| Non-smoker | 57 | 27.0 | 15 | 21.7 | 72 | 25.7 | 115 | 35.0 |
| Missing | 35 | 16.6 | 13 | 18.8 | 48 | 17.1 | 15 | 4.6 |
| Tobacco type |  |  |  |  |  |  |  |  |
| Chewing Tobacco | 3 | 1.4 | 0 | 0 | 3 | 1.1 | 2 | 0.6 |
| Cigarettes | 107 | 50.7 | 41 | 59.4 | 148 | 52.9 | 191 | 58.1 |
| Cigars | 0 | 0 | 0 | 0 | 0 | 0 | 1 | 0.3 |
| Other | 6 | 2.8 | 0 | 0 | 6 | 2.1 | 4 | 1.2 |
| Snuff | 2 | 1.0 | 0 | 0 | 2 | 0.7 | 0 | 0 |
| Missing | 93 | 44.1 | 28 | 40.6 | 121 | 43.2 | 131 | 39.8 |
| Height (cm) |  |  |  |  |  |  |  |  |
| Mean ±SD | 169.4 ±9.6 | – | 170.2 ±15.1 | – | 170.0 ±9.5 | – | 169.8 ±11.7 | – |
| Median (range) | 169 (143–197.5) | – | 173 (71.5–188) | – | 170.0 (143–197.5) | – | 169.5 (63–198) | – |
| Weight (kg) |  |  |  |  |  |  |  |  |
| Mean ±SD | 75.9 ±20.2 | – | 78.2 ± 20.3 | – | 76.5 ± 20.2 | – | 77.4 ±19.2 | – |
| Median (range) | 73 (37.4–160.4) | – | 74.4 (43.8–146) | – | 73.6 (37.4–160.4) | – | 75.3 (39.9–192.0) | – |
| BMI (kg/m^2^) |  |  |  |  |  |  |  |  |
| Mean ±SD | 27.5 ±18.8 | – | 27.9 ±13.5 | – | 26.3 ±6.0 | – | 27.1 ±10.0 | – |
| Median (range) | 25.9 (12.9–283.5) | – | 24.9 (16.2–126.9) | – | 25.6 (12.9–50.1) | – | 25.8 (15.1–176.1) | – |

*Note* Percentages may not add up to 100 because of rounding.

Abbreviation: BMI, body mass index; ECOG PS, Eastern Cooperative Oncology group performance status; NOS, not otherwise specified.

^a^Baseline not true included patients who completed a baseline assessment after, but within 3 weeks of, initiating first-line therapy.

**Supplemental Table S4 |** Means and standard deviations of MDASI symptom severity and interference by assessment timepoints 0 to 30 weeks (*N* = 609).

| **Time Points** | **0_week(baseline), *n* = 280** | | **6_week,**  ***n* = 294** | | **12_week,**  ***n* = 287** | | **18_week,**  ***n* = 252** | | **24_week,**  ***n* = 236** | | **30_week,**  ***n* = 225** | |
| --- | --- | --- | --- | --- | --- | --- | --- | --- | --- | --- | --- | --- |
|  | mean | sd | mean | sd | mean | sd | mean | sd | mean | sd | mean | sd |
| **Symptoms** |  | | | | | | | | | | | |
| Pain | 3.03 | 3.17 | 2.38 | 2.94 | 1.92 | 2.74 | 1.90 | 2.65 | 2.27 | 2.84 | 2.19 | 2.86 |
| Fatigue | 3.95 | 3.14 | 3.59 | 2.98 | 3.26 | 2.87 | 3.42 | 3.01 | 3.09 | 2.84 | 3.25 | 2.87 |
| Nausea | 1.00 | 2.22 | 1.06 | 2.24 | 0.95 | 2.03 | 0.81 | 1.74 | 0.75 | 1.86 | 0.88 | 2.02 |
| Disturbed sleep | 2.92 | 3.30 | 2.32 | 2.90 | 2.05 | 2.91 | 1.97 | 2.81 | 2.16 | 2.86 | 2.14 | 2.87 |
| Distress | 2.17 | 2.74 | 1.49 | 2.49 | 1.26 | 2.10 | 1.34 | 2.26 | 1.58 | 2.35 | 1.48 | 2.51 |
| Shortness of breath | 3.09 | 3.11 | 2.31 | 2.94 | 2.17 | 2.73 | 2.34 | 2.90 | 2.11 | 2.70 | 1.96 | 2.50 |
| Remember | 1.51 | 2.37 | 1.64 | 2.36 | 1.60 | 2.32 | 1.54 | 2.41 | 1.49 | 2.29 | 1.69 | 2.30 |
| Appetite | 2.23 | 2.93 | 2.05 | 2.76 | 1.68 | 2.65 | 1.79 | 2.75 | 1.46 | 2.32 | 1.62 | 2.57 |
| Drowsiness | 2.70 | 2.93 | 2.17 | 2.55 | 1.94 | 2.58 | 1.85 | 2.66 | 1.88 | 2.52 | 1.67 | 2.25 |
| Dry mouth | 2.39 | 3.08 | 2.19 | 2.81 | 1.97 | 2.84 | 2.14 | 2.80 | 1.69 | 2.54 | 1.89 | 2.59 |
| Sad | 1.44 | 2.15 | 1.12 | 2.07 | 0.85 | 1.79 | 1.04 | 2.04 | 1.17 | 2.27 | 1.07 | 2.08 |
| Vomiting | 0.32 | 1.22 | 0.39 | 1.56 | 0.36 | 1.41 | 0.29 | 1.12 | 0.25 | 1.18 | 0.33 | 1.45 |
| Numbness | 1.06 | 2.23 | 1.06 | 2.19 | 0.98 | 1.95 | 1.09 | 2.12 | 1.26 | 2.45 | 1.41 | 2.45 |
| Coughing | 2.52 | 2.92 | 1.87 | 2.57 | 1.69 | 2.41 | 1.70 | 2.42 | 1.58 | 2.31 | 1.51 | 2.18 |
| Constipation | 1.72 | 2.67 | 1.82 | 2.73 | 1.36 | 2.41 | 1.38 | 2.54 | 1.15 | 2.29 | 1.25 | 2.20 |
| Sore throat | 0.73 | 1.85 | 0.57 | 1.63 | 0.40 | 1.25 | 0.51 | 1.34 | 0.39 | 1.21 | 0.49 | 1.45 |
| **Interference Items** |  | | | | | | | | | | | |
| Work | 3.25 | 3.65 | 3.10 | 3.34 | 2.26 | 3.01 | 2.50 | 3.07 | 2.27 | 2.91 | 2.26 | 3.20 |
| Activity | 3.69 | 3.51 | 3.03 | 3.18 | 2.50 | 3.03 | 2.64 | 3.15 | 2.46 | 2.97 | 2.48 | 3.13 |
| Walking | 3.08 | 3.36 | 2.60 | 3.16 | 1.90 | 2.74 | 2.15 | 2.99 | 1.76 | 2.65 | 1.88 | 2.89 |
| Enjoy life | 2.86 | 3.31 | 2.09 | 2.90 | 1.60 | 2.58 | 1.61 | 2.56 | 1.45 | 2.42 | 1.88 | 2.87 |
| Mood | 2.25 | 2.74 | 1.89 | 2.52 | 1.52 | 2.34 | 1.66 | 2.57 | 1.48 | 2.42 | 1.58 | 2.40 |
| Relations | 1.24 | 2.21 | 1.15 | 2.07 | 0.80 | 1.60 | 0.97 | 2.07 | 0.94 | 1.91 | 0.71 | 1.76 |
| **Symptom Subscale** |  | | | | | | | | | | | |
| Severity top 5^a^ | 3.14 | 2.31 | 2.56 | 2.08 | 2.27 | 2.03 | 2.29 | 2.05 | 2.30 | 1.96 | 2.24 | 1.96 |
| Total severity (13core +3 module) | 2.05 | 1.53 | 1.75 | 1.43 | 1.53 | 1.39 | 1.57 | 1.37 | 1.52 | 1.32 | 1.55 | 1.39 |
| Total core severity (13 items) | 2.14 | 1.67 | 1.83 | 1.49 | 1.62 | 1.48 | 1.66 | 1.48 | 1.63 | 1.46 | 1.66 | 1.52 |
| Total LC module severity (3 module items) | 1.65 | 1.59 | 1.42 | 1.65 | 1.15 | 1.47 | 1.20 | 1.47 | 1.04 | 1.38 | 1.08 | 1.33 |
| **Interference Subscale** |  | | | | | | | | | | | |
| Total interference | 2.73 | 2.55 | 2.31 | 2.31 | 1.76 | 2.08 | 1.93 | 2.24 | 1.73 | 2.05 | 1.80 | 2.26 |
| WAW (work/activity/walking) | 3.34 | 3.07 | 2.91 | 2.91 | 2.22 | 2.59 | 2.43 | 2.67 | 2.17 | 2.56 | 2.21 | 2.80 |
| REM (relations/enjoylife/mood) | 2.12 | 2.37 | 1.71 | 2.12 | 1.31 | 1.88 | 1.41 | 2.17 | 1.29 | 1.90 | 1.39 | 2.02 |

^a^The top 5 most severe symptoms were fatigue, pain, disturbed sleep, shortness of breath, and dry mouth).

**Supplemental Table S5 |** Means and standard deviations of MDASI symptom severity and interference by assessment timepoints 36 to 66 weeks (*N* = 609).

| **Time Points** | **36_week,**  ***n* = 222** | | **42_week,**  ***n* = 190** | | **48_week,**  ***n* = 186** | | **54_week,**  ***n* = 180** | | **60_week,**  ***n* = 164** | | **66_week,**  ***n* = 156** | |
| --- | --- | --- | --- | --- | --- | --- | --- | --- | --- | --- | --- | --- |
|  | mean | sd | mean | sd | mean | sd | mean | sd | mean | sd | mean | sd |
| **Symptoms** |  | | | | | | | | | | | |
| Pain | 2.05 | 2.64 | 2.03 | 2.72 | 2.25 | 2.89 | 2.38 | 2.91 | 2.02 | 2.53 | 2.20 | 3.00 |
| Fatigue | 3.06 | 2.92 | 2.93 | 2.94 | 3.18 | 2.68 | 3.21 | 2.82 | 2.91 | 2.67 | 3.12 | 2.92 |
| Nausea | 0.76 | 1.81 | 0.70 | 1.60 | 0.65 | 1.58 | 0.80 | 1.85 | 0.80 | 1.78 | 0.75 | 1.80 |
| Disturbed sleep | 2.18 | 2.89 | 1.33 | 2.06 | 1.93 | 2.58 | 1.78 | 2.42 | 1.63 | 2.28 | 1.90 | 2.67 |
| Distress | 1.29 | 2.29 | 1.42 | 2.25 | 1.18 | 2.06 | 1.22 | 2.03 | 1.10 | 1.88 | 1.12 | 1.87 |
| Shortness of breath | 1.86 | 2.61 | 1.82 | 2.36 | 1.88 | 2.69 | 1.80 | 2.37 | 1.72 | 2.35 | 1.77 | 2.55 |
| Remember | 1.36 | 2.20 | 1.40 | 2.06 | 1.31 | 2.14 | 1.43 | 2.18 | 1.40 | 1.97 | 1.50 | 2.35 |
| Appetite | 1.48 | 2.67 | 1.23 | 2.30 | 1.35 | 2.42 | 1.25 | 2.12 | 1.14 | 2.09 | 1.58 | 2.57 |
| Drowsiness | 1.92 | 2.50 | 1.19 | 2.02 | 1.53 | 2.16 | 1.24 | 2.00 | 1.35 | 1.84 | 1.57 | 2.40 |
| Dry mouth | 2.03 | 2.94 | 1.61 | 2.43 | 2.03 | 2.90 | 1.56 | 2.44 | 1.51 | 2.33 | 1.66 | 2.39 |
| Sad | 1.04 | 1.98 | 0.76 | 1.57 | 0.82 | 1.82 | 1.03 | 2.11 | 0.67 | 1.65 | 0.81 | 1.70 |
| Vomiting | 0.43 | 1.54 | 0.20 | 1.01 | 0.25 | 1.15 | 0.26 | 1.34 | 0.23 | 0.94 | 0.41 | 1.53 |
| Numbness | 0.93 | 1.85 | 0.93 | 1.80 | 1.27 | 2.34 | 1.01 | 1.72 | 1.46 | 2.36 | 1.23 | 2.21 |
| Coughing | 1.41 | 2.23 | 1.41 | 2.28 | 1.41 | 2.23 | 1.10 | 1.72 | 1.42 | 2.05 | 1.60 | 2.66 |
| Constipation | 1.30 | 2.39 | 1.14 | 2.28 | 1.16 | 2.13 | 0.86 | 1.79 | 1.31 | 2.26 | 1.21 | 2.25 |
| Sore throat | 0.41 | 1.40 | 0.32 | 1.17 | 0.35 | 1.13 | 0.33 | 1.25 | 0.23 | 0.75 | 0.40 | 1.37 |
| **Interference Items** |  | | | | | | | | | | | |
| Work | 2.16 | 2.90 | 2.22 | 3.16 | 2.13 | 2.95 | 1.97 | 2.83 | 1.63 | 2.52 | 2.34 | 3.09 |
| Activity | 2.24 | 3.01 | 2.27 | 3.17 | 2.12 | 2.93 | 1.98 | 2.86 | 1.86 | 2.81 | 2.06 | 3.02 |
| Walking | 2.03 | 2.83 | 1.97 | 2.97 | 1.95 | 2.95 | 1.89 | 2.78 | 1.67 | 2.60 | 1.88 | 2.70 |
| Enjoy life | 1.56 | 2.61 | 1.27 | 2.27 | 1.37 | 2.28 | 1.04 | 2.15 | 1.04 | 2.10 | 0.94 | 2.09 |
| Mood | 1.30 | 2.24 | 1.49 | 2.42 | 1.24 | 2.09 | 1.24 | 2.02 | 1.09 | 1.92 | 1.17 | 2.36 |
| Relations | 0.72 | 1.77 | 0.62 | 1.57 | 0.63 | 1.68 | 0.67 | 1.67 | 0.28 | 0.84 | 0.56 | 1.59 |
| **Symptom Subscale** |  | | | | | | | | | | | |
| Severity top 5^a^ | 2.22 | 2.05 | 1.86 | 1.71 | 2.16 | 1.82 | 2.08 | 1.78 | 1.93 | 1.59 | 2.11 | 1.91 |
| Total severity (13core +3 module) | 1.47 | 1.41 | 1.28 | 1.14 | 1.41 | 1.24 | 1.33 | 1.22 | 1.31 | 1.06 | 1.43 | 1.21 |
| Total core severity (13 items) | 1.57 | 1.54 | 1.35 | 1.21 | 1.51 | 1.35 | 1.46 | 1.33 | 1.38 | 1.14 | 1.51 | 1.30 |
| Total LC module severity (3 module items) | 1.04 | 1.37 | 0.96 | 1.32 | 0.98 | 1.26 | 0.76 | 1.17 | 1.00 | 1.14 | 1.07 | 1.40 |
| **Interference Subscale** |  | | | | | | | | | | | |
| Total interference | 1.67 | 2.08 | 1.64 | 2.09 | 1.58 | 2.01 | 1.47 | 1.93 | 1.26 | 1.69 | 1.49 | 1.99 |
| WAW (work/activity/walking) | 2.14 | 2.59 | 2.15 | 2.80 | 2.07 | 2.70 | 1.96 | 2.57 | 1.72 | 2.38 | 2.10 | 2.66 |
| REM (relations/enjoylife/mood) | 1.20 | 1.91 | 1.13 | 1.80 | 1.09 | 1.79 | 0.98 | 1.72 | 0.80 | 1.34 | 0.89 | 1.81 |

^a^The top 5 most severe symptoms were fatigue, pain, disturbed sleep, shortness of breath, and dry mouth).

**Supplemental Table S6 |** Means and standard deviations of MDASI symptom severity and interference by assessment timepoints 72 to 102 weeks (*N* = 609).

| **Time Points** | **72_week,**  ***n* = 138** | | **78_week,**  ***n* = 124** | | **84_week,**  ***n* = 119** | | **90_week,**  ***n* = 104** | | **96_week,**  ***n* = 100** | | **102_week,**  ***n* = 96** | |
| --- | --- | --- | --- | --- | --- | --- | --- | --- | --- | --- | --- | --- |
|  | mean | sd | mean | sd | mean | sd | mean | sd | mean | sd | mean | sd |
| **Symptoms** |  | | | | | | | | | | | |
| Pain | 2.01 | 2.87 | 2.04 | 2.67 | 1.88 | 2.83 | 1.68 | 2.42 | 1.72 | 2.45 | 1.38 | 2.35 |
| Fatigue | 3.17 | 2.95 | 3.00 | 2.82 | 2.48 | 2.81 | 2.63 | 2.82 | 2.92 | 3.07 | 2.10 | 2.50 |
| Nausea | 0.73 | 1.93 | 0.72 | 1.76 | 0.58 | 1.32 | 0.63 | 1.57 | 0.50 | 1.58 | 0.34 | 1.14 |
| Disturbed sleep | 1.76 | 2.47 | 1.69 | 2.56 | 1.78 | 2.75 | 1.77 | 2.73 | 1.21 | 2.23 | 1.32 | 2.16 |
| Distress | 0.94 | 1.91 | 0.99 | 2.00 | 0.70 | 1.87 | 0.97 | 2.08 | 0.65 | 1.81 | 0.71 | 1.56 |
| Shortness of breath | 1.91 | 2.46 | 1.72 | 2.36 | 1.47 | 2.25 | 1.40 | 2.14 | 1.31 | 2.24 | 1.70 | 2.36 |
| Remember | 1.52 | 2.33 | 1.52 | 2.34 | 1.18 | 2.24 | 1.34 | 2.00 | 1.21 | 1.71 | 1.09 | 2.05 |
| Appetite | 1.48 | 2.80 | 1.26 | 2.25 | 0.98 | 2.15 | 0.97 | 1.85 | 1.03 | 2.32 | 0.89 | 1.96 |
| Drowsiness | 1.33 | 2.21 | 1.40 | 2.30 | 1.40 | 2.24 | 1.26 | 2.05 | 1.22 | 2.18 | 1.18 | 2.00 |
| Dry mouth | 2.15 | 2.89 | 1.47 | 2.39 | 1.68 | 2.58 | 1.54 | 2.42 | 1.27 | 2.03 | 1.79 | 2.63 |
| Sad | 0.72 | 1.76 | 0.67 | 1.63 | 0.47 | 1.73 | 0.43 | 1.18 | 0.57 | 1.51 | 0.43 | 1.63 |
| Vomiting | 0.33 | 1.34 | 0.30 | 1.20 | 0.24 | 1.31 | 0.24 | 1.06 | 0.10 | 0.61 | 0.24 | 1.20 |
| Numbness | 1.27 | 2.28 | 1.45 | 2.29 | 0.94 | 1.79 | 1.11 | 1.91 | 1.05 | 2.36 | 1.37 | 2.37 |
| Coughing | 1.50 | 2.36 | 1.43 | 2.23 | 1.04 | 1.95 | 1.42 | 2.34 | 1.52 | 2.38 | 1.10 | 1.92 |
| Constipation | 1.40 | 2.42 | 1.12 | 2.30 | 0.86 | 1.86 | 0.91 | 2.04 | 1.43 | 2.98 | 0.91 | 1.86 |
| Sore throat | 0.42 | 1.23 | 0.30 | 1.12 | 0.53 | 1.61 | 0.42 | 1.40 | 0.24 | 1.05 | 0.28 | 1.15 |
| **Interference Items** |  | | | | | | | | | | | |
| Work | 2.36 | 3.13 | 2.25 | 3.05 | 1.46 | 2.31 | 1.09 | 1.95 | 1.49 | 2.65 | 1.33 | 2.33 |
| Activity | 2.29 | 3.06 | 2.18 | 2.84 | 1.61 | 2.42 | 1.27 | 1.92 | 1.49 | 2.64 | 1.16 | 2.22 |
| Walking | 2.18 | 3.14 | 1.72 | 2.76 | 1.34 | 2.34 | 0.81 | 1.68 | 1.21 | 2.45 | 1.07 | 2.29 |
| Enjoy life | 1.49 | 2.82 | 0.97 | 2.04 | 0.89 | 1.89 | 0.69 | 1.53 | 0.50 | 1.44 | 0.51 | 1.31 |
| Mood | 1.57 | 2.60 | 1.07 | 2.08 | 0.59 | 1.43 | 0.89 | 1.58 | 0.73 | 1.81 | 0.84 | 1.96 |
| Relations | 1.03 | 2.32 | 0.54 | 1.58 | 0.27 | 1.07 | 0.30 | 0.86 | 0.31 | 1.34 | 0.18 | 0.86 |
| **Symptom Subscale** |  | | | | | | | | | | | |
| Severity top 5^a^ | 2.04 | 1.93 | 1.97 | 1.75 | 1.80 | 1.93 | 1.75 | 1.75 | 1.68 | 1.75 | 1.54 | 1.56 |
| Total severity (13core +3 module) | 1.42 | 1.36 | 1.32 | 1.19 | 1.14 | 1.21 | 1.17 | 1.13 | 1.12 | 1.22 | 1.05 | 1.03 |
| Total core severity (13 items) | 1.49 | 1.48 | 1.40 | 1.25 | 1.21 | 1.33 | 1.23 | 1.19 | 1.14 | 1.22 | 1.12 | 1.09 |
| Total LC module severity (3 module items) | 1.11 | 1.36 | 0.95 | 1.31 | 0.80 | 1.26 | 0.91 | 1.32 | 1.06 | 1.59 | 0.76 | 1.20 |
| **Interference Subscale** |  | | | | | | | | | | | |
| Total interference | 1.83 | 2.44 | 1.45 | 1.90 | 1.03 | 1.47 | 0.85 | 1.20 | 0.96 | 1.36 | 0.85 | 1.39 |
| WAW (work/activity/walking) | 2.27 | 2.94 | 2.05 | 2.70 | 1.47 | 2.09 | 1.05 | 1.61 | 1.40 | 2.21 | 1.19 | 2.02 |
| REM (relations/enjoy life/mood) | 1.40 | 2.44 | 0.86 | 1.54 | 0.58 | 1.24 | 0.63 | 1.15 | 0.52 | 1.12 | 0.51 | 1.17 |

^a^The top 5 most severe symptoms were fatigue, pain, disturbed sleep, shortness of breath, and dry mouth.

**Supplemental TABLE S7** | Numbers and percentages of patients in each parameter grouping for the mixed-effects models (*N* = 609).

|  | **N** | **%** |
| --- | --- | --- |
| **Best 1L treatment response (clinician-graded)** |  |  |
| Complete response or partial response | 200 | 32.8 |
| Stable disease | 118 | 19.4 |
| Progressive disease | 170 | 27.9 |
| Missing | 121 | 19.9 |
| **Treatment type** |  |  |
| Chemo and chemo+biologic | 117 | 19.2 |
| Chemo+immuno and chemo+biologic+immuno | 232 | 38.1 |
| Targeted therapy and targeted therapy+biologic | 176 | 28.9 |
| Immunotherapy | 82 | 13.5 |
| Biologic | 2 | 0.3 |

Abbreviations: 1L, first-line therapy; chemo, chemotherapy.

**Supplemental Table S8 |** Summary of statistically significant mixed-effects modeling results.

| **Analysis** | **Outcome** | **Statistically Significant Result (*P*< 0.05)** | ***P*-value** |
| --- | --- | --- | --- |
| **MDASI symptom severity by best 1L treatment clinician-graded response** | Fatigue | Progressive disease associated with more severity than complete/partial response | <0.001 |
|  | Pain | Progressive disease associated with more severity than complete/partial response | <0.001 |
|  | Shortness of breath | Progressive disease associated with more severity than complete/partial response | <0.001 |
|  | Disturbed sleep | Progressive disease associated with more severity than complete/partial response | <0.001 |
|  | Feeling drowsy | Progressive disease associated with more severity than complete/partial response | <0.001 |
|  | Dry mouth | Progressive disease associated with more severity than complete/partial response | <0.001 |
|  | Coughing | Progressive disease associated with more severity than complete/partial response | <0.001 |
|  | Lack of appetite | Progressive disease associated with more severity than complete/partial response | <0.001 |
|  | Trouble remembering | Progressive disease associated with more severity than complete/partial response | 0.036 |
|  | Constipation | Progressive disease associated with more severity than complete/partial response | <0.001 |
|  | Feeling distressed | Progressive disease associated with more severity than complete/partial response | <0.001 |
|  | Feeling sad | Progressive disease associated with more severity than complete/partial response | <0.001 |
|  | Nausea | Progressive disease associated with more severity than complete/partial response | <0.001 |
|  | Sore throat | Progressive disease associated with more severity than complete/partial response | 0.007 |
|  | Vomiting | Progressive disease associated with more severity than complete/partial response | 0.002 |
| **MDASI symptom interference by best 1L treatment clinician-graded response** | General activity | Progressive disease associated with more interference than complete/partial response | <0.001 |
|  | Work | Progressive disease associated with more interference than complete/partial response | <0.001 |
|  | Walking | Progressive disease associated with more interference than complete/partial response | <0.001 |
|  | Enjoyment of life | Progressive disease associated with more interference than complete/partial response | <0.001 |
|  | Mood | Progressive disease associated with more interference than complete/partial response | <0.001 |
|  | Relations with others | Progressive disease associated with more interference than complete/partial response | <0.001 |
| **MDASI symptom severity by 1L treatment type** | Pain | Target therapy associated with less severity than chemotherapy | <0.001 |
|  | Shortness of breath | Target therapy associated with less severity than chemotherapy | <0.001 |
|  | Feeling drowsy | Target therapy associated with less severity than chemotherapy | 0.015 |
|  | Coughing | Target therapy associated with less severity than chemotherapy | 0.004 |
|  | Trouble remembering | Target therapy associated with less severity than chemotherapy | 0.038 |
|  | Numbness or tingling | Target therapy associated with less severity than chemotherapy | 0.001 |
| **MDASI symptom interference by 1L treatment type** | General activity | Target therapy associated with less interference than chemotherapy | 0.013 |
|  | Work | Target therapy associated with less interference than chemotherapy | 0.002 |
|  | Walking | Target therapy associated with less interference than chemotherapy | 0.001 |
| **EQ VAS by best 1L treatment clinician-graded response** | Overall health rating | Progressive disease associated with lower overall health rating than complete/partial response | <0.001 |
| **EQ-VAS by 1L treatment type** | Overall health rating | Targeted therapy associated with higher overall health rating than chemotherapy | 0.002 |

**References**

1. Pickard AS, Law EH, Jiang R, et al. United States valuation of EQ-5D-5L health states using an international protocol. *Value Health.* 2019;22(8):931-941. doi:10.1016/j.jval.2019.02.009.

2. EuroQol Group. EQ-5D-5L. https://euroqol.org/eq-5d-instruments/eq-5d-5l-about/. Accessed November 21, 2024.
